# Antioxidant Effects of *Turbo cornutus* By-Products Visceral Extract against Hydrogen Peroxide-Induced Oxidative Stress by Regulating MAPK and Akt Signaling Pathways in Vero Cells

**DOI:** 10.3390/foods12193660

**Published:** 2023-10-04

**Authors:** Yeon-Ji Lee, Eun-A Kim, Nalae Kang, Areumi Park, Soo-Jin Heo

**Affiliations:** 1 Jeju Bio Research Center, Korea Institute of Ocean Science and Technology (KIOST), Jeju 63349, Republic of Korea; leeyj0409@kiost.ac.kr (Y.-J.L.); euna0718@kiost.ac.kr (E.-A.K.); nalae1207@kiost.ac.kr (N.K.); areumi1001@kiost.ac.kr (A.P.); 2 Department of Marine Technology & Convergence Engineering (Marine Biotechnology), University of Science and Technology (UST), Daejeon 34113, Republic of Korea

**Keywords:** *Turbo cornutus*, visceral tissue, by-products, taurine, antioxidant effect, Vero cells, oxidative stress

## Abstract

*Turbo cornutus*, a marine gastropod mollusk commonly called sea snail, is found along the southern coast of Korea and holds considerable importance as a marine food resource, particularly on Jeju Island, Korea. Data are scarce on the antioxidant activity of hot water extracts from *T. cornutus* visceral tissue. Therefore, this study was performed to evaluate the antioxidant activities of *T*. *cornutus* visceral tissue hot water extract (TVE) and the underlying mechanisms against hydrogen peroxide-induced oxidative stress in Vero cells. The amino acid composition and antioxidant effects of TVE were evaluated. Furthermore, the impact of TVE on the expression of proteins within the mitogen-activated protein kinase (MAPK) pathway is investigated. TVE showed a concentration-dependent enhancement in its scavenging activities against 2,2-diphenyl-1-picrylhydrazyl (DPPH) radicals (IC_50_ = 1.07 ± 0.06 mg/mL) and hydrogen peroxide (IC_50_ = 0.33 ± 0.03 mg/mL). TVE reduced intracellular reactive oxygen species (ROS) production and maintained cell viability under H_2_O_2_-induced oxidative stress by suppressing apoptosis in Vero cells. Additionally, TVE demonstrated regulatory effects on the MAPK and protein kinase B (Akt) signaling pathways activated by H_2_O_2_. In conclusion, the findings from our study propose that TVE holds potential as a bioactive component in the formulation of functional foods.

## 1. Introduction

*Turbo cornutus*, a marine gastropod mollusk, also known as sea snail, is distributed along the southern coast of Korea. *T*. *cornutus* found on Jeju Island is important in the food industry as a strategic local specialty. The total production of *T*. *cornutus* in Republic of Korea was estimated to be 2376 tons in 2020 [1]. As large quantities of *T. cornutus* are produced each year, the *T*. *cornutus* visceral tissue and shells are discarded after processing due to low consumer preference and awareness [2]. Gastropod-derived foods exhibit many beneficial properties such as anti-inflammatory and antioxidant properties [2,3,4].

Oxidative stress is caused by a disturbance in the balance between reactive oxygen species (ROS) production and antioxidant defenses [5]. Excessive ROS production can damage lipids, proteins, and DNA and is associated with the development of various diseases, including vascular disorders, autoimmune diseases, neurodegenerative diseases, and respiratory diseases [6]. To prevent ROS accumulation, cellular systems involve antioxidant mechanisms such as mitogen-activated protein kinase (MAPK) and protein kinase B (Akt)-mediated signaling pathways [7,8].

Previous studies have reported that Kim et al. [3] demonstrated the anti-inflammatory effect of an ethanolic extract of *T*. *cornutus* viscera on lipopolysaccharide-stimulated RAW 264.7 cells and a zebrafish model. And the hydrolysates of *T*. *cornutus* viscera have potential antioxidant and bioactive peptide properties in the human hepatoblastoma (HepG2) cell line [2]. In addition, abalone (*Haliotis discus* hannai) viscera hydrolysates, a species of gastropod, have antioxidant activity and antioxidant amino acids [4]. As therapeutic candidates, peptides have been researched and applied in pharmaceutical fields such as drug discovery and antibacterial peptides [9]. Although hot water extraction is an efficient technique for extracting bioactive compounds in the food industry [10], studies on the antioxidant activity of the hot water extract of *T. cornutus* visceral tissue are lacking.

This study aims to investigate whether *T*. *cornutus* visceral tissue hot water extract (TVE) exhibited antioxidant activity that could ameliorate hydrogen peroxide (H_2_O_2_)-induced oxidative stress in Vero cells.

## 2. Materials and Methods

### 2.1. Sample Collection and Extraction

*T*. *cornutus* was obtained from a fishing village in Udo, Republic of Korea. The shells of *T*. *cornutus* were removed, and the muscle and visceral tissues were separated and washed with tap water. Muscle and visceral tissues were lyophilized and ground into a powder. Hot water or enzyme-assisted extraction is being used for the application of various natural resources for food and nutraceutical purposes [11]. Extraction was performed using distilled water via three methods: (1) For traditional extraction, a freeze-dried *T*. *cornutus* sample (2 g) was extracted with distilled water (100 mL) using a shaking incubator for 24 h at room temperature. (2) Artificial digestion for extraction of *T*. *cornutus* was performed according to the method described by Oh et al. [12]. (3) For hot water extraction, 2 g of freeze-dried sample was pressure-extracted with 100 mL of distilled water at 121 °C for 20 min. After extraction using each method, the extract was centrifuged at 3200× *g* rpm for 10 min and filtered through a filter paper (Whatman, Maidstone, Kent, United Kingdom). The filtrate was lyophilized and stored at refrigerator temperatures (−20 °C) until used in experiments.

### 2.2. Analysis of Chemical Composition

Bicinchoninic acid (BCA) method, a copper-based colorimetric assay, was used to determine the total protein content in TVE while using bovine serum albumin as the reference standard. Briefly, BCA protein reagents were added to samples and then reacted for 30 min at 37 °C. Absorbance was measured at 562 nm using a microplate reader (Multiskan; Thermo Fisher Scientific, Waltham, MA, USA). Carbohydrate content was analyzed by modifying the methods described by Han et al. [13] using D-glucose as the reference standard.

### 2.3. Measurement of Amino Acid

High-performance liquid chromatography (HPLC)-fluorescence detection system has been widely used for quantitative amino acid analysis [14]. The samples for amino acid analysis were hydrolyzed with 6 N hydrogen chloride following the method described by Kang et al. [2]. The amino acid composition of TVE was determined using an HPLC system (Ultimate 3000; Thermo-Dionex, Sunnyvale, CA, USA) consisting of a fluorescence detector (1260FLD; Agilent Technologies, Santa Clara, CA, USA). Amino acids were determined using an inno C18 column (5 μm, 4.6 × 150 mm; YoungJin Biochrom, Seongnam-si, Gyeoggi-do, Republic of Korea). The experimental conditions were as follows: mobile phase (water:acetonitrile:methanol = 10:45:45); flow rate (1.5 mL/min); and column temperature (40 °C).

### 2.4. Availability of Antioxidant Activity

Freeze-dried samples were dissolved in distilled water at various concentrations for antioxidant assays. To measure the antioxidant activity of TVE, DPPH radical scavenging and H_2_O_2_-scavenging assays were performed using modified methods of Chatatikun and Chiabchalard [15] and Kim et al. [16], respectively. The half-maximal inhibitory concentration (IC_50_) values were obtained.

### 2.5. Cell Culture

Vero cells (African green monkey kidney cells) were purchased from the Korean Cell Line Bank (KCLB, Seoul, Republic of Korea). Cells were incubated at 37 °C with 5% CO_2_ in RPMI 1640 containing 10% fetal bovine serum (FBS), penicillin (10,000 Units/mL), and streptomycin (10 mg/mL). Cells were subcultured or seeded upon reaching 80–90% confluency for experiments.

### 2.6. Measurement of Cell Viability

TVE cytotoxicity was evaluated using the 3-(4,5-Dimethylthiazol-2-yl)-2,5-diphenyl-tetrazolium bromide (MTT) assay as described by Kim et al. [3]. The cells were seeded in a 96-well plate at a density of 1 × 10^5^ cells/mL and incubated for 24 h. To determine the cytoprotective effect of TVE, the cells were pre-incubated with 25, 50, 100, and 200 μg/mL TVE for 1 h before stimulation with 200 μM H_2_O_2_. After 24 h, the MTT reagent was added to each well for 3 h, and the formazan produced was dissolved using dimethyl sulfoxide (DMSO). The absorbance was measured at 540 nm using a microplate reader (BioTek Synergy HT; Agilent Technologies, Santa Clara, CA, USA).

### 2.7. Evaluation of Intracellular ROS Level

The cells were seeded in a 96-well plate at a density of 1 × 10^5^ cells/mL and incubated for 24 h. The cells were then pre-treated with 25, 50, 100, and 200 μg/mL TVE for 1 h before stimulation with 200 μM H_2_O_2_. After 30 min, the DCFH-DA reagent was added. 2′,7′-dichlorodihydrofluorescein diacetate (DCFH-DA) fluorescence was measured using a microplate reader (BioTek Synergy HT; Agilent Technologies, Santa Clara, CA, USA) and used to calculate the intracellular ROS levels.

### 2.8. Apoptosis Analysis

Nuclear morphology was confirmed using Hoechst 33342 (NucBlue™ Live ReadyProbes™ Reagent, Invitrogen, Waltham, MA, USA), a cell-permeable DNA-staining sample. Vero cells were seeded in a four-well chamber slide (Sigma-Aldrich, St. Louis, MO, USA) and incubated for 24 h. The cells were pretreated with TVE for 1 h and stimulated with H_2_O_2_ (200 μM) for 6 h. Nuclei were stained with Hoechst 33342 solution according to the manufacturer’s specifications. The stained cells were observed under a fluorescence microscope.

### 2.9. Western Blotting

The cells were seeded in 60 mm dishes for 24 h and then pre-treated with different concentrations of TVE for 1 h; all dishes, except the control, were subsequently stimulated with H_2_O_2_ for 10 min. Cells were washed twice with phosphate-buffered saline, harvested, washed, and lysed in radioimmunoprecipitation assay buffer. The protein content of the cell lysates was measured using a BCA protein assay kit (Thermo Fisher Scientific, Waltham, MA, USA). Equal volumes of lysates were electrophoresed on 4–12% Bis-Tris protein gels and transferred to nitrocellulose membranes. Primary antibodies used were as follows: anti-phosphorylated (phospho)-p38, anti-p38, anti-phospho-extracellular signal-regulated kinase (ERK), anti-ERK, anti-phospho-c-Jun N-terminal kinase (JNK), anti-JNK, anti-phospho-Akt, anti-Akt, and anti-β-actin. The secondary antibodies used were anti-rabbit and anti-mouse IgG. Antibodies were purchased from Cell Signaling Technology (Beverly, MA, USA), Santa Cruz Biotechnology (Dallas, TX, USA), and Invitrogen (Thermo Fisher Scientific, Waltham, MA, USA). Proteins were detected using a chemiluminescence detection kit (EZ-Western Lumi Pico; DoGen Bio, Seoul, Republic of Korea) on a Davinch–Chemi Imaging System (CAS400SM; Davinch-K, Seoul, Republic of Korea). Expression levels were quantified using ImageJ version 1.53t (National Institutes of Health, Bethesda, MD, USA).

### 2.10. Statistical Analysis

All experiments were performed in triplicate. Statistical significance was assessed using one-way ANOVA followed by Dunnett’s multiple comparison test using GraphPad Prism software version 9 (GraphPad Software, San Diego, CA, USA), and *p* values < 0.05 were considered significant.

## 3. Results and Discussion

### 3.1. H_2_O_2_-scavenging Activity of Various T. cornutus Extracts

**Figure 1:**
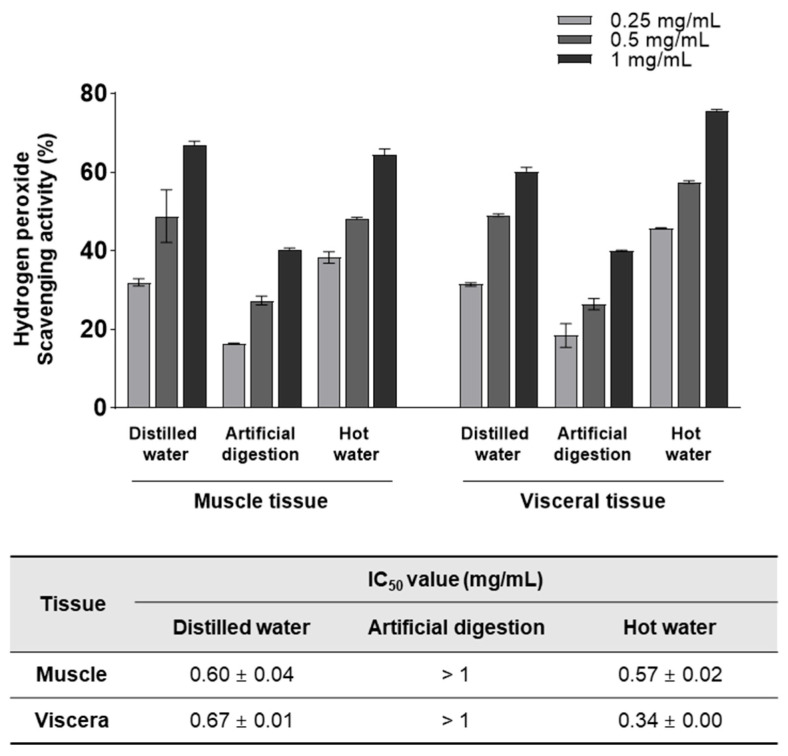
Hydrogen peroxide-scavenging activity of various *T*. *cornutus* extracts. The hydrogen peroxide-scavenging activity was evaluated in the muscle and visceral tissues of *T*. *cornutus* extracts using three extraction methods. Data represent the mean ± standard deviation from triplication. IC_50_, half-maximal inhibitory concentration.

The yield and activity of the extracts depend significantly on the solvent used, temperature, pressure, and extraction time [17]. To investigate the impact of various water-based extraction methods on the H_2_O_2_-scavenging effect, extraction techniques using distilled water, artificial digestion, and hot water were selected. The overall H_2_O_2_-scavenging activity of *T*. *cornutus* hot water extracts was higher than that of the distilled water and artificial digestion extracts (Figure 1). In addition, in the hot water extract, the H_2_O_2_-scavenging activity of the visceral tissue-derived extract (IC_50_ = 0.34 ± 0.00 mg/mL) was higher than that of the muscle tissue-derived extract (IC_50_ = 0.57 ± 0.02 mg/mL). These results showed that TVE had the highest antioxidant activity. Therefore, subsequent experiments were performed using TVE.

### 3.2. Extraction Yield and Proximate Composition of TVE

The composition of TVE is presented in Table 1. The extraction yield of TVE was 37.31 ± 0.41%, which was plentiful protein (41.08 ± 1.01%) and carbohydrate (19.69 ± 1.70%). Han et al. [13] have found that the hydrolysate from the gastropod Batillus cornutus meat tissue has a protein content of 37.94 ± 0.17%. A comparison of these results showed that TVE had a similar protein content. Therefore, it is considered that TVE will be an excellent protein source.

### 3.3. Amino Acid Composition in TVE

It was confirmed that the main component of TVE is protein. Therefore, TVE analyzed amino acids, which are constituents of proteins. The amino acid composition of TVE is presented in Table 2. The most abundant amino acid was taurine (2-aminoethanesulfonic acid), accounting for 40.75 ± 1.65% of the total amino acid pool. Additionally, TVE was abundant in glutamic acid (10.98 ± 0.75%), aspartic acid (7.01 ± 0.00%), and glycine (6.65 ± 1.00%). The results confirmed that TVE is a good source of taurine. Previous studies reported that the alkaline protease hydrolysate derived from octopuses is rich in taurine (23.16%) and exhibits antioxidant activity [18]. Taurine is a non-proteinogenic sulfur-containing amino acid that exerts cytoprotective effects [19]. Taurine has several biological functions including anti-inflammatory, antioxidant, vasodilatory, and anti-apoptotic effects [20,21]. These results demonstrated that TVE contained higher amounts of taurine with various biological activities than octopus hydrolysate. Seafood is an excellent source of taurine, which improves human health by exerting cytoprotective actions via various mechanisms, including antioxidants, energy production, neuromodulation, Ca^2+^ homeostasis, and osmotic regulation [22].

### 3.4. Antioxidant Activities of TVE

**Figure 2:**
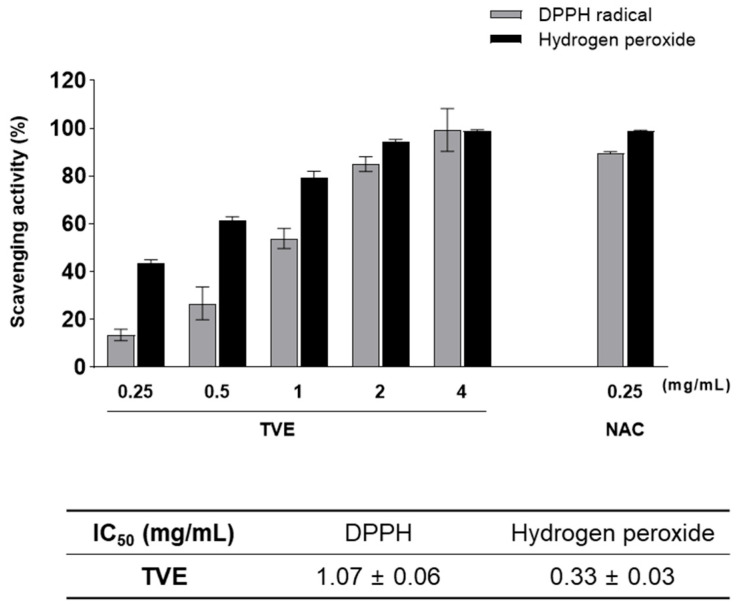
Availability of antioxidant activity of TVE. DPPH radical and hydrogen peroxide−scavenging activity of TVE with reference to NAC as a positive control. Data represent the mean ± standard deviation from triplication. TVE, *Turbo cornutus* visceral tissue hot water extract; NAC, *N*-acetyl cysteine; IC_50_, half-maximal inhibitory concentration.

The antioxidant activity of TVE was measured using DPPH radical and H_2_O_2_-scavenging assays and compared to that of the positive control, NAC (Figure 2). TVE showed a concentration-dependent increase in DPPH radical and H_2_O_2_-scavenging activities with IC_50_ values of 1.07 ± 0.06 and 0.33 ± 0.03 mg/mL, respectively. The DPPH radical and H_2_O_2_-scavenging effects of 4 mg/mL TVE (99.35 ± 8.96 and 98.93 ± 0.52%, respectively) were similar to those of NAC (89.8 ± 0.5 and 98.78 ± 0.45%, respectively).

Kang et al. [2] reported that *T. cornutus* viscera protamex-assisted extracts had H_2_O_2_-scavenging activity with an IC_50_ value of 0.435 mg/mL. It has been demonstrated that TVE possessed higher H_2_O_2_-scavenging than *T. cornutus* viscera hydrolysate.

These results indicated that TVE has excellent inhibitory activity against DPPH radicals and H_2_O_2_.

### 3.5. Effect of TVE on Cell Viability and ROS Generation in H_2_O_2_-Stimulated Vero Cells

**Figure 3:**
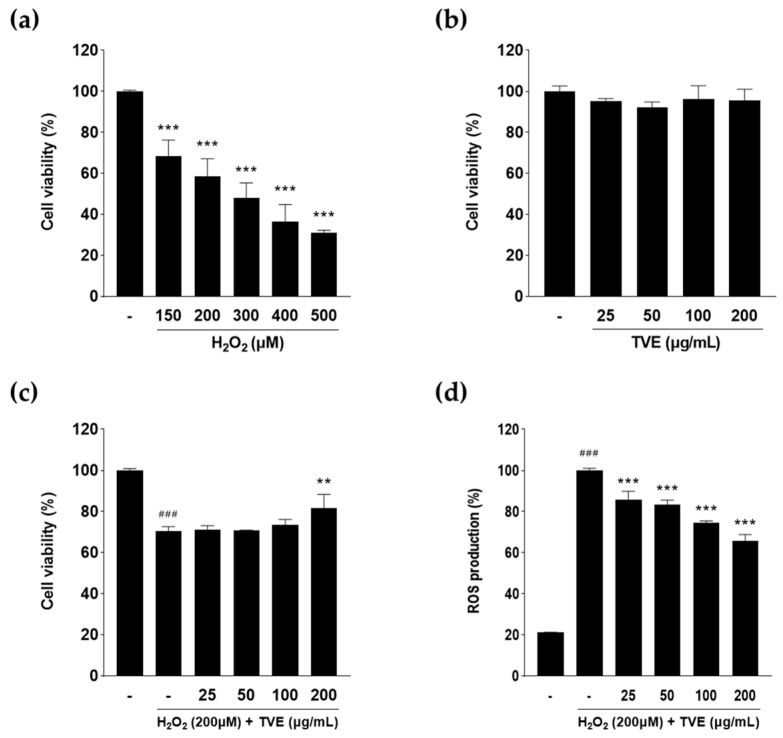
Protective effect of TVE against H_2_O_2_−induced oxidative stress in Vero cells. (**a**) Toxicity in H_2_O_2_−treated Vero cells. Cells were incubated for 24 h with various concentrations of H_2_O_2_ (150−500 μM). (**b**) Viability of TVE−treated Vero cells. Cells were cultured for 24 h with various concentrations of TVE (0−200 μg/mL). (**c**) Protective effect of TVE in H_2_O_2_−treated cells. (**d**) ROS generation in H_2_O_2_−treated cells after treatment with TVE. Cells were treated for 24 h with TVE (0−200 μg/mL) in the presence of H_2_O_2_ (200 μM). Data represent the mean ± standard deviation from triplication. ^###^ *p* < 0.001 as compared control group, ** *p* < 0.01, *** *p* < 0.001 as compared with the H_2_O_2_−treated group. TVE, *Turbo cornutus* visceral tissue hot water extract; ROS, reactive oxygen species; H_2_O_2_, hydrogen peroxide.

Based on the antioxidant activity of TVE, further experiments were performed to evaluate the viability and intracellular ROS levels in H_2_O_2_-stimulated Vero cells. H_2_O_2_ at a concentration of 200 μM reduced cell viability to approximately 58.35 ± 8.83%, which was considered optimal for inducing cytotoxicity in the subsequent experiments (Figure 3A). TVE at concentrations ranging from 25 to 200 μg/mL was not cytotoxic to Vero cells; further experiments were conducted using these concentrations (Figure 3B). H_2_O_2_ stimulation reduced cell viability and increased intracellular ROS levels compared to the control (Figure 3C,D). TVE increased cell viability and reduced intracellular ROS levels in H_2_O_2_-stimulated cells in a concentration-dependent manner. The intracellular ROS levels in cells pre-treated with 25, 50, 100, and 200 μg/mL TVE were 85.8 ± 4.0, 83.3 ± 2.2, 74.5 ± 0.9, and 65.6 ± 3.2%, respectively. These results indicated that TVE exerts a cytoprotective effect against H_2_O_2_-induced oxidative stress.

### 3.6. Effect of TVE on H_2_O_2_-Induced Apoptosis in Vero Cells

**Figure 4:**
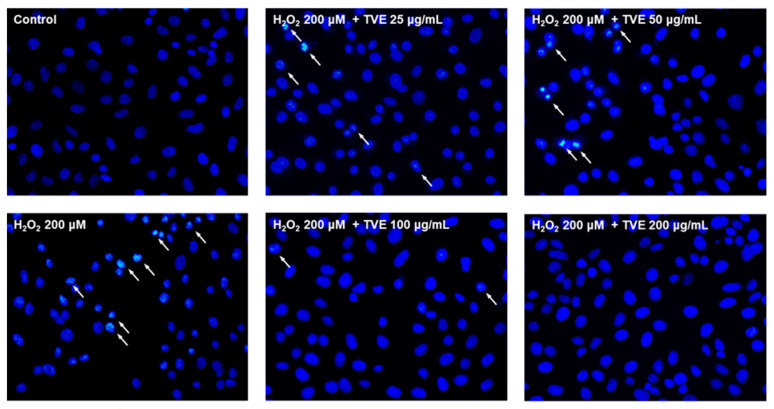
Anti-apoptotic effects of TVE in H_2_O_2_−stimulated Vero cells. Cells were pre-treated with TVE (0−200 μg/mL) for 1 h and then stimulated with H_2_O_2_ (200 μM). After incubation for 6 h, the cells were stained with Hoechst 33342. Representative morphology was observed under a fluorescence microscope (Eclipse 80i). Apoptotic cells (arrows) containing condensed and fragmented nuclei exhibited bright fluorescence. TVE, *T*. *cornutus* visceral tissue hot water extract; H_2_O_2_, hydrogen peroxide.

ROS overproduction can activate cell death processes such as apoptosis [23]. Hoechst 33342 is taken up by both dead and live cells, and it is used to detect chromatin condensation and DNA fragmentation in apoptotic cells [24]. H_2_O_2_ treatment increased apoptotic body formation, as observed by nuclear condensation and fragmentation, according to Hoechst 33342 staining (Figure 4). TVE treatment suppressed apoptotic body formation in a concentration-dependent manner. The results suggested that TVE prevents H_2_O_2_-induced cell death by suppressing apoptosis via ROS scavenging.

### 3.7. Effect of TVE on Regulation of MAPK and Akt Signaling Pathways

**Figure 5:**
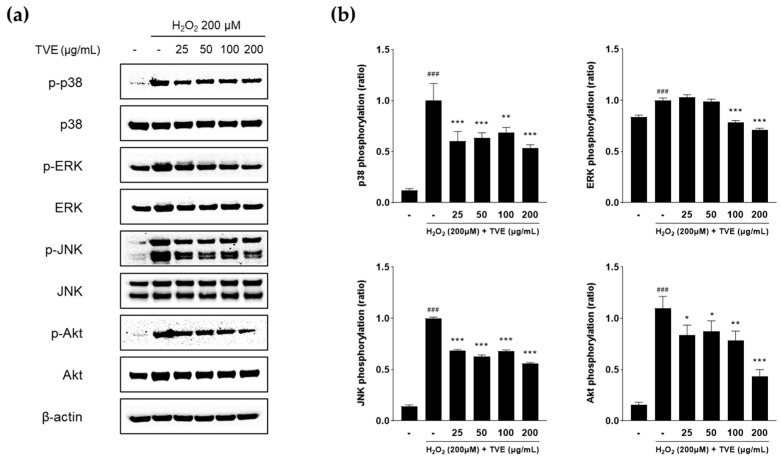
Effect of TVE on H_2_O_2_−induced MAPK and Akt protein expression. (**a**) The protein expression of total and phosphorylated MAPKs and Akt in H_2_O_2_−induced Vero cells was measured via Western blotting and β-actin was used as a loading control. (**b**) Relative protein expression of phosphorylated proteins in H_2_O_2_−stimulated Vero cells. ImageJ software was used to determine the intensity of the bands. Data represent the mean ± standard deviation from triplication. ^###^ *p* < 0.001 as compared to the control group; * *p* < 0.05, ** *p* < 0.01, *** *p* < 0.001 as compared with the H_2_O_2_−treated group. TVE, *T*. *cornutus* visceral tissue hot water extract; H_2_O_2_, hydrogen peroxide; MAPK, mitogen-activated protein kinase.

Antioxidants can scavenge ROS and reduce the oxidation of cellular molecules [25]; ROS play vital roles in mediating MAPK and Akt activation [8]. MAPK and Akt play essential roles in transmitting cell signals, consequently influencing the expression of genes that regulate various cellular processes such as cell growth, proliferation, and apoptosis [26]. To investigate the molecular mechanisms underlying the antioxidant effects of TVE, the levels of phosphorylated MAPKs, including p38, ERK, JNK, and Akt, were analyzed via Western blotting. H_2_O_2_ treatment markedly increased the phosphorylation of p38, ERK, JNK, and Akt (Figure 5). In addition, TVE pretreatment attenuated the phosphorylation of MAPK and Akt signaling. These results indicated that TVE protects cells against H_2_O_2_-induced oxidative stress by inhibiting the MAPK and Akt signaling pathways.

## 4. Conclusions

The visceral tissue of *T*. *cornutus* is considered a post-harvest by-product. Several studies have reported that visceral tissues of *T. cornutus* have antioxidant and anti-inflammatory properties. The present study demonstrated that TVE, a taurine-rich protein source, protected cells against H_2_O_2_-induced oxidative stress by scavenging intracellular ROS and inhibiting apoptosis via deactivation of the MAPK and Akt pathways in Vero cells. In the future, additional experiments are needed, such as how taurine, an amino acid abundant in TVE, acts on cytoprotection, ROS generation, and antioxidant mechanisms. In conclusion, this study suggests that *T*. *cornutus* visceral tissue, a marine by-product, can be used for developing various functional food ingredients and may provide potential health benefits to humans.

## Figures and Tables

**Table 1:** Extraction yield and composition of TVE.

Sample	Yield (%)	Proximate Composition (%)	
		Protein	Carbohydrate
TVE ^1^	37.31 ± 0.41	41.08 ± 1.01	19.69 ± 1.70

TVE, *Turbo cornutus* visceral tissue hot water extract. ^1^ All values are presented as the mean ± standard deviation (*n* = 3).

**Table 2:** Amino acid composition of TVE.

Amino Acid	TVE ^1^
	% Amino Acid
Taurine	40.75 ± 1.65
Aspartic acid	7.01 ± 0.00
Glutamic acid	10.98 ± 0.75
Serine	3.12 ± 0.05
Histidine	1.43 ± 0.07
Glycine	6.65 ± 1.00
Threonine	3.03 ± 0.36
Arginine	4.57 ± 0.26
Alanine	3.46 ± 0.21
Tyrosine	1.53 ± 0.04
Valine	2.50 ± 0.14
Methionine	1.10 ± 0.09
Phenylalanine	2.27 ± 0.10
Isoleucine	1.86 ± 0.02
Leucine	3.12 ± 0.05
Lysine	1.82 ± 0.31
Proline	4.80 ± 0.27
Total	100.00

TVE, *Turbo cornutus* visceral tissue hot water extract. ^1^ All values are presented as the mean ± standard deviation (*n* = 3).

## Data Availability

The data presented in this study are available upon request from the corresponding author.
